# Overview of the retina and imaging in patients with severe acute respiratory syndrome coronavirus 2

**DOI:** 10.1007/s10792-022-02338-x

**Published:** 2022-05-11

**Authors:** Solmaz Abdolrahimzadeh, Manuel Lodesani, Daria Rullo, Alberto Mariani, Gianluca Scuderi

**Affiliations:** 1 Ophthalmology Unit, Neurosciences, Mental Health, and Sense Organs (NESMOS) Department, Sapienza University of Rome, Rome, Italy; 2 Faculty of Medicine and Psychology, St. Andrea Hospital, Via di Grottarossa 1035/1039, 00189 Rome, Italy

**Keywords:** Coronavirus, COVID-19, SARS-CoV-2, Fundus examination, Spectral domain optical coherence tomography, Optical coherence tomography angiography, Retina

## Abstract

**Introduction:**

The role of the human eye in severe acute respiratory syndrome coronavirus 2 (SARS-COV-2) is still under investigation. The pathophysiology of the ocular findings is arduous when dealing with critically ill Covid-19 patients with comorbidities. Multiorgan involvement and the effects of inflammation, infection and systemic treatment on the retina are complex, and comparison of studies is difficult. Most studies in human patients have investigated the anterior segment, whereas few reports deal with the posterior segment of the eye. The present review aims to evaluate the retinal manifestations and imaging features in COVID-19 patients.

**Methods:**

Studies on the retinal manifestations and retinal imaging in COVID-19 patients published through June 2021 were reviewed. We included cross-sectional and case–control studies, case series, case reports and correspondence in the analysis.

**Results:**

Flame-shaped hemorrhages, cotton wool spots, augmented diameter and tortuosity of retinal vessels were found on funduscopic examination. Peripapillary, macular retinal nerve fiber layer and ganglion cell layer thickness alterations were reported on spectral domain optical coherence tomography. Reduced vessel density of the superficial and deep retinal capillary plexus on optical coherence tomography angiography was reported.

**Conclusions:**

Retinal complications may arise in COVID-19 patients. Although no consensus on presentation is currently available, retinal funduscopy and imaging has shown neuronal and vascular alterations. Systemic neurological complications and microangiopathy are associated with SARS-COV-2; thus, as the retina has a neuronal and vascular component, funduscopy and retinal imaging on COVID-19 patients can provide further insight to SARS-COV-2 disease and the follow-up of patients.

## Introduction

In December 2019 an outbreak of pneumonia occurred in Wuhan, China. The cause at the time was still unknown, but following health authority investigations, a novel coronavirus causing severe pneumonia was isolated. Further genomic research identified the virus as severe acute respiratory syndrome coronavirus 2 (SARS-CoV-2) that is principally replicated and propagated in the respiratory system leading to an immune response [1]. Since 2019 the SARS-COV-2 virus has gone through adaptive mutation in the viral genome that could result in increase in transmissibility, increase in virulence or change in clinical disease presentation. Even though numerous variants of SARS-COV-2 have been described, only a few are considered variants of concern such as the Delta variant, identified in December 2020 and responsible for the second wave of COVID-19 infections and the recent Omicron variant, identified in South Africa in November 2021, and currently cause of a spike in the number of infections [2]. The role of the eye in the SARS-CoV-2 pandemic is still under investigation, and the major body of research refers to the 2019 virus variant.

Ocular infection by coronaviruses has been well established in the animal model. There is evidence of sight-threatening ocular complication in the feline and murine orders [3]. In feline coronavirus it has been suggested that the ocular manifestations are due to a vasculitis and inflammation leading to conjunctivitis, anterior uveitis, choroiditis and retinal vasculitis [4]. Experimental studies on coronavirus retinopathy with intravitreal inoculation of virus from JHMV-infected mice showed an initial inflammatory response followed by retinal degeneration [5]. The pathological mechanism initiates with localization of the virus in the retina and retinal pigment epithelium and causes inflammation owing to pro-inflammatory mediator release. Although viral clearance occurs after a week from infection, the secondary autoimmune response with production of retinal and retinal pigment epithelium autoantibodies leads to neuroretinal thinning and loss of the photoreceptors and ganglion cells [6].

Cellular infection of SARS-CoV-2 occurs through binding of the viral spike protein to the cellular angiotensin-converting enzyme 2 (ACE2), and serine protease TMPRSS2 promotes spike protein priming [7, 8]. In humans ACE2 protein is expressed in epithelial cells in the lungs and other tissues such as the intestine and kidney [1]. In the human eye these proteins are expressed in the cornea, conjunctiva and iris, but ACE2 is also detectable in the aqueous humor and retina [8, 9]. In particular, ACE and ACE2 are found in the ganglion cells, Muller cells, photoreceptors, retinal vascular cells and the choroid [10]. Casagrande et al. analyzed retinal biopsies of 14 patients deceased for confirmed SARS-CoV-2 infection, and three of 14 samples were positive for the SARS-CoV-2 RdRp-gene, E-gene and Orf nCoV-gene-specific sequences in the retina [11]. Moreover, retinal microvascular alterations and vascular occlusions have been reported [12].

The virus can reach the retina through various possible mechanisms. Raony et al. suggested a hematogenous route to the central nervous system [13], and Figueiredo et al. proposed spread of the virus through direct infection of capillary endothelial cells expressing ACE2 and CD147 or via leukocytes that cross the blood retinal barrier carrying the virus to the retina [14]. In the neuronal route, spreading could occur through retrograde transportation from the brain to the optic nerve and the retinal ganglion cells [14]. Furthermore, some authors suggested that the corneal nerves can spread the virus from the anterior segment toward other regions via the trigeminal nerve [15, 16]. However, few reports have evaluated the manifestations of the posterior segment of the eye. Potentially, the status of the retina, evaluated with ophthalmoscopic examination and imaging, can provide indications on systemic disease. In this review, we analyze publications relative to retinal alterations and imaging with spectral domain optical coherence tomography (SDOCT) and optical coherence tomography angiography (OCTA) in patients diagnosed with COVID-19.

## Methods

**Fig. 1:**
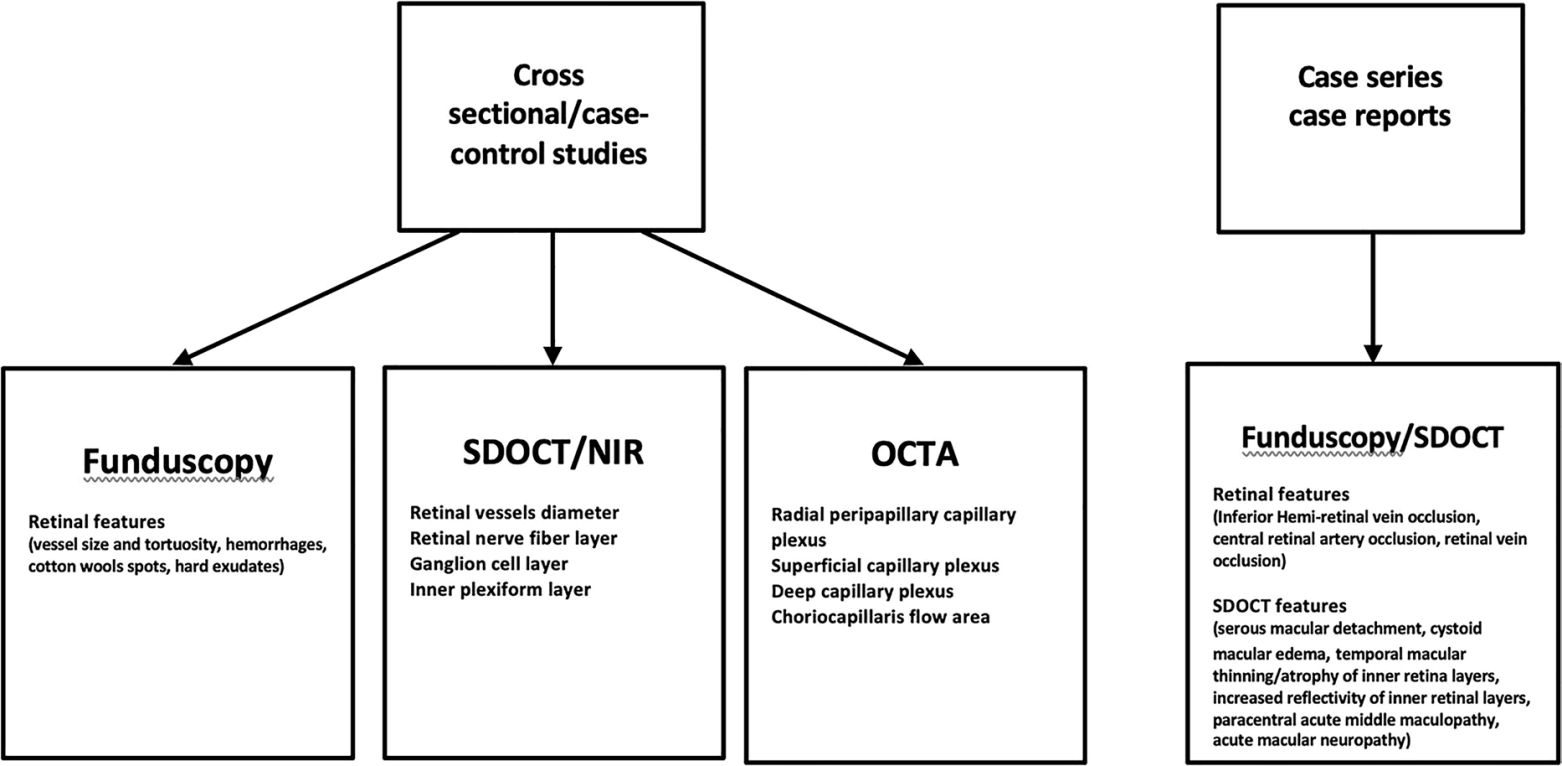
Flowchart of publications analyzed for retinal alterations and imaging features in COVID-19 patients. The parameters evaluated in fundus examination, spectral domain optical coherence tomography (SDOCT) and optical coherence tomography angiography (OCTA) are summarized

The literature search was carried out from the PubMed, Web of Science and Scopus electronic databases through June 2021. The keywords used were “COVID-19” OR “SARS-CoV-2” OR “2019-nCoV” AND “retinal” OR “fundus” OR “imaging.” Reference lists of included articles and pertinent reviews were also searched. Three authors (ML, DR, AM) independently screened all the titles and abstracts and reference lists of all included articles. Full manuscripts of relevant articles were evaluated by all authors and subjected to group discussion. The flowchart in figure one shows the retinal features evaluated in the publications included in the study (Fig. 1).

## Results

We included cross-sectional and case–control studies, case series, case reports and correspondence on the retinal alterations and retinal imaging features in confirmed COVID-19 patients. We excluded any reports with only suspected cases. Editorials and narrative reviews were also excluded. Data specifically included COVID-19 positivity, systemic clinical manifestations and therapy, retinal signs and complications, and retinal imaging features of COVID-19 patients.

Flame-shaped hemorrhages, cotton wool spots, augmented diameter and tortuosity of retinal vessels were found on funduscopic examination. Peripapillary, macular retinal nerve fiber layer and ganglion cell layer thickness alterations were reported on SDOCT. Reduced vessel density of the superficial and deep retinal capillary plexus on OCTA was reported.

## Discussion

The pathophysiology of the ocular and retinal findings appears arduous when dealing with critically ill COVID-19 patients with comorbidities. Patients have multiorgan involvement with coagulopathy, sepsis, fluid overload and hypo-oxygenation [17–22]. The effects of infection and inflammation, systemic treatment including antibodies, steroids, vasopressors, ventilation, heparinization or anticoagulation, and dialysis on the retina are so complex that comparison of studies is difficult. However, ocular infection by coronaviruses is well established in the feline and murine orders and in humans, retinal alterations and specific imaging features in COVID-19 patients have been reported in the literature.

### Fundus alterations

Various studies have demonstrated the correlation between SARS-CoV-2 infection and an increase of coagulation abnormalities [17, 18]. One major cause of the systemic thromboembolic and inflammatory complications of COVID-19 seems to be endothelial damage [19–21]. The vascular status of the retina, evaluated with ophthalmoscopic examination, provides an indication of the systemic vascular system. Similar to other systemic diseases, such as diabetes and hypertension, evaluation of the retina can potentially provide indications of alterations linked to COVID-19.

There are some case reports of retinal occlusive disease in patients with SARS-CoV-2. Montesel et al. described central artery occlusion in a patient and hypothesized a multifactorial process of inflammation and hypercoagulation as risk factors [22]. Sheth et al. described hemiretinal vein occlusion [23] and Invernizzi et al. reported a case of central retinal vein occlusion in a patient with multiple retinal hemorrhages, increased venular tortuosity and a diffuse fern-like retinal whitening [24]. Marinho et al. found microhemorrhages and cotton wool spots in a case series of 4 patients [25].

Studies with larger cohorts of patients successively revealed somewhat contrasting results. Pirraglia et al. reported on 43 SARS-CoV-2 patients with pneumonia but did not find signs of posterior segment involvement [26]. However, the patients in their cohort were treated with low molecular weight heparin to prevent systemic vascular complications. Pereira et al. performed fundus photography in 18 hospitalized patients with severe COVID-19 and found flame-shaped hemorrhages, cotton wool spots, peripheral retinal hemorrhages, retinal epithelium hyperplasia, macular hemorrhages and hard exudates in 10 patients, suggesting acute vascular lesions of the inner retina [27]. These authors hypothesized that these alterations could be due to damage to neuronal tissue, similar to other viral diseases that have a systemic and retinal impact such as HIV, dengue and Zika virus [28, 29]. Invernizzi et al. studied 54 patients with COVID-19 and 133 control subjects. They assessed fundus photographs and evaluated mean artery diameter (MAD) and mean vein diameter (MVD). Retinal alterations in patients with COVID-19 were dilated veins (27.7%), tortuous vessels (12.9%), hemorrhages (9.25%) and cotton wools spots (7.4%). The authors found that both MAD and MVD were higher in COVID-19 patients compared to controls and MVD was positively correlated with disease in severe and non-severe cases compared to unexposed subjects [30]. Nevertheless, in this study there was a high prevalence of hypertension and diabetes in the cohort; thus, the authors suggested that the data should be interpreted with caution.

To date, reports in the literature indicate that posterior segment alterations can be associated with SARS-CoV-2. However, direct comparison is limited as the available studies are not uniform in design. The contrasting results could be due to patient selection where severity of disease, pre-existing systemic conditions other than COVID-19 and therapies administered are variable. Sen et al., in a recent systematic review, reported that posterior ocular manifestations can present from one week to six weeks after the onset of COVID-19 symptomatology. Retinal hemorrhages and cotton wool spots are the most common but do not affect visual acuity; however, retinal vein occlusion with macular edema and rarely retinal arterial occlusions and ocular inflammation are sight-threatening complications [31].

### Spectral domain optical coherence tomography and optical coherence tomography angiography

Some authors used SDOCT to study posterior segment alterations in patients with COVID-19. The rationale is linked to the neurological alterations such as anosmia, ageusia, encephalopathy, headache, ataxia, epileptic seizures and cerebrovascular disease that are described in 30–40% of patients [32, 33]. Thus, as the retina has neuronal and vascular components, examination with SDOCT and OCTA could provide further insight to the disease.

Case series studies have shown SDOCT alterations in COVID-19 patients. Invernizzi et al. found hyperreflectivity of the inner retinal layers in a patient with impending retinal vein occlusion [24]. Marinho et al. described hyperreflective lesions of the ganglion cell layer (GCL) and inner plexiform layer (IPL) in proximity of the papillomacular bundle. They suggested that these findings could be associated with the central nervous system manifestations described in COVID-19 patients [25]. Virgo and Mohamed observed focal volume loss of the inner nuclear layer and corresponding hyperreflectivity in the inner and outer plexiform layers consistent with diagnosis of paracentral acute middle maculopathy (PAMM) in one patient and interdigitation zone disruption with outer plexiform layer hyperreflectivity consistent with diagnosis of acute macular neuroretinopathy (AMN) in another case [34]. The authors hypothesized postinfection complications linked to inflammation and ischemia, as PAMM and AMN have been reported to be associated with febrile conditions [35] and reduced retinal vascular flow [36, 37].

Recently larger cohort studies were published with data on retinal layer thickness and vascular density using SDOCT and OCTA. Burgos-Blasco et al. investigated the thickness of the peripapillary retinal fiber layer (RNFL), macular RNFL, GCL and IPL in 90 patients recovered from COVID 19 and compared results to 70 control subjects. The study group presented increased global RNFL thickness and increased superior and inferior nasal peripapillary RNFL thickness. In contrast, macular RNFL thickness was decreased in COVID-19 patients in the superior inner, nasal inner, and nasal outer quadrants. The GCL thickness was increased in volume, in the superior, nasal and inferior outer quadrants. Peripapillary RNFL and macular GCL thickness was increased in patients with anosmia and ageusia [38]. The authors suggested that the thickening of neuronal retinal layers could be due to acute damage of the virus to the optic nerve that, in later stages, could potentially lead to atrophy similar to that reported in SDOCT evaluation of Parkinson patients [39, 40]. Although the exact mechanism for these changes in SARS-CoV-2 infection is not clear, a trans-synaptic retrograde mechanism originating from the olfactory bulb has been advanced [11, 41].

Savastano et al. reported a reduction of the radial peripapillary capillary plexus density in 80 recovered COVID-19 patients and compared this with healthy control subjects using OCTA. The perfusion density correlated with lopinavir/ritonavir or antiplatelet therapy [42]. However, in contrast to the results of Burgos et al. [38], Savastano et al. did not find differences in average RNFL thickness between patients and healthy controls. The authors found a linear correlation of RNFL thickness and retinal peripapillary vascular density in COVID-19 patients [42].

Indeed, OCTA is an interesting line of research that enables the study of the choriocapillaris and retinal vascular density. As these two structures have the highest vascularization for area unit in the human body, Turker et al. speculated that the consequences of COVID-19 could be studied in a non-invasive and rapid manner using OCTA and that the microangiopathy that characterizes COVID-19 infection can potentially cause changes in the vascular density of the retina and the choriocapillaris [43]. These authors reported OCTA findings in 54 eyes of 27 patients compared with healthy controls. The foveal and parafoveal vascular density was reduced in the superior and nasal quadrants of the superficial capillary plexus and globally reduced in the deep capillary plexus in the study group. Furthermore, COVID-19 patients had significantly higher choriocapillaris flow area values compared with controls. The authors speculated that this could be due to vasodilatation of the choriocapillaris vessels as a reaction to hypoxia, ischemia or inflammation, favoring the theory that the systemic ischemic effects of SARS-CoV-2, including the presence of the virus in the retina, could be the basis for microvascular alterations [43].

Abrishami et al. used OCTA to assess foveal and parafoveal vascular density and the extent of the foveal avascular zone (FAZ) in 31 infected patients compared to 23 healthy controls. They found a significant reduction of mean vessel density in the superficial capillary plexus and deep capillary plexus of the retina in the COVID-19 cohort. Moreover, they described an increased FAZ in the COVID-19 group, but these data were not statistically significant. The authors speculated that this reduction could be related to both direct coronavirus infection of the retina and secondary inflammatory effects [44].

A comprehensive summary of the retinal alterations and imaging of the posterior ocular segment in the current literature is shown in Table 1.

**Table 1:** Summary of studies describing the posterior segment of the eye in severe acute respiratory syndrome coronavirus 2

Author	Patients	Eyes	Age	Preexisting disease	Patient status	Systemic therapy	Fundus alterations	SDOCT	OCTA
Pirraglia et al. [26]	43	86	70	Hypertension (n22), diabetes (n8), CAD (n7), COPD (n7), CVA/TIA (n6), PAD (n5), CHF (n4), AF (n4), dementia (n4), hemiplegia (n2), liver disease (n2)	Hospitalized	Hydroxychloroquine, azithromycin tocilizumab, weight-based low-molecular-weight heparin, steroids	**–**	**–**	**–**
Invernizzi et al. [30]	54	108	49.9	Hypertension (n16), diabetes (n8), HIV (n3), TB (n2), alcohol consumption (n12), dyslipidemia (n7), CAD/stroke (n5)	Hospitalized	Oxygen therapy, anticoagulant, antiaggregant, hydroxychloroquine, remdesivir, lopinavir/ritonavir, tocilizumab, steroids	Hemorrhages (n5), cotton wools spots (n4), dilated veins (n15), tortuous vessels (n7)	NIR to assess retinal vessels diameter	/
Marinho et al. [25]	12	24	25–69	–	Hospitalized (n2), discharged (n10)	–	Cotton wool spots (n4), Microhemorrhages (n4)	GCL/IPL hyper-reflectivity (n4)	Normal
Pereira et al. [27]	18	36	62.5	Hypertension (n12), diabetes (n9)	Hospitalized in ICU (n17)	Invasive mechanical ventilation (n14), vasoactive pharmacological support (n8), prophylactic anticoagulation therapy (n8), full-intensity anticoagulation therapy (n7)	Flame-shaped hemorrhages (n5), cotton wool spots (n4), retinal sectorial pallor (n1), peripheral retinal hemorrhages (n2), retinal pigment epithelium hyperplasia (n1), choroidal naevus (n1), macular hemorrhages and hard exudates (n1)	–	–
Burgos Blasco et al. [38]	90	180	55.5	Hypertension (n26), diabetes (n8), dyslipidemia (n25)	Hospitalized	–	Normal	Increased peripapillary RNFL/decreased macular RNLF thickness	–
Savastano et al. [42]	80	160	52.9	–	Discharged (5 pts previously in ICU)	Hydroxychloroquine (n55), lopinavir + ritonavir (n27), darunavir + ritonavir (n35), azithromycin (n8), heparin (n33), antiplatelet therapy (n6), steroids (n4)	–	RNFL average thickness linearly correlated to RPCP flow index and RPCP perfusion density	Pts with systemic arterial hypertension: lower RPCP flow index Age inversely correlated to RPCP flow index and RPCP perfusion density Patients treated with lopinavir + ritonavir or antiplatelet: reduced RPCP flow index and RPCP perfusion density
Turker et al. [43]	27	59	38.7	–	Discharged	–	–	–	Decreased parafoveal SCP /DCP vessels density, increased CC flow area
Abrishami et al. [44]	31	62	40.4	–	Hospitalized (n9), out patients (n22)	Oxygen therapy in hospitalized patients (n6)	–	Normal	Decreased SCP/DCP vessel density
Case reports									
Sheth et al. [23]	1	1	52	–	Discharged	–	Inferior Hemi-RVO, tortuous retinal veins	Serous macular detachment, cystoid macular edema	–
Virgo et al. [34]	2	2	34.5	Acephalgic visual migraine aura and right toxoplasma chorioretinitis (n1), acephalgic visual migraine aura (n1)	–	–	Normal	PAMM (n1), AMN (n1)	–
Montesel et al. [22]	1	1	59	Hypertension, hyperuricemia	Discharged (previously in ICU)	Mechanical ventilation, hydroxychloroquine, lopinavir/ritonavir, IV tocilizumab	CRAO	Temporal macular thinning (both eyes), atrophy of inner retina layers/loss of foveal depression (one eye)	–
Invernizzi et al. [24]	1	1	54	–	Out patient	Steroids	Hemorrhages, perivenular whitening	Increased reflectivity of middle retinal layers	–

*AF* atrial fibrillation; *AMN* acute macular neuroretinopathy; *CAD* coronary artery disease; *CC* choriocapillaris; *CHF* congestive heart failure; *COPD* chronic obstructive pulmonary disease; *CRAO* central retinal artery occlusion; *CVA* cerebrovascular accident; *DCP* deep capillary plexus; *GCL* ganglion cell layer; *HIV* human immunodeficiency virus; *ICU* intensive care unit; *ipl* inner plexiform layer; *NIR* near-infrared reflectance; *OCTA* optical coherence tomography angiography; *PAD* peripheral artery disease; *PAMM* paracentral acute middle maculopathy; *Pts* patients; *RNFL* retinal nerve fiber layer; *RPCP* radial peripapillary capillary plexus; *RVO* retinal vein occlusion; *SCP* superficial capillary plexus; *SDOCT* spectral domain optical coherence tomography; *TB* tuberculosis; *TIA* transient ischemic attack

## Conclusions

The studies analyzed in this review show that there are alterations of the vascular and neuronal components of the retina related to SARS-COV-2 infection. Direct comparison of the studies currently available in the literature is difficult owing to different study designs that included patients in various stages of disease with variable treatment modalities. Given that the eye is a window on numerous systemic diseases characterized by neuronal and vascular alterations, retinal examination, SDOCT, and OCTA imaging can provide further insight on the pathogenetic mechanisms of disease and possibly aid in the follow-up of patients with SARS-COV-2 infection.

## Data Availability

Not applicable.
